# Building better lithium-sulfur batteries: from LiNO_3_ to solid oxide catalyst

**DOI:** 10.1038/srep33154

**Published:** 2016-09-15

**Authors:** Ning Ding, Lan Zhou, Changwei Zhou, Dongsheng Geng, Jin Yang, Sheau Wei Chien, Zhaolin Liu, Man-Fai Ng, Aishui Yu, T. S. Andy Hor, Michael B. Sullivan, Yun Zong

**Affiliations:** 1 Institute of Materials Research and Engineering (IMRE), A*STAR (Agency for Science, Technology and Research), 2 Fusionopolis Way, Innovis #08-03, Singapore 138634, Republic of Singapore; 2 Department of Chemistry, Shanghai Key Laboratory of Molecular Catalysis and Innovative Materials, Institute of New Energy, Fudan University, Shanghai 200438, P.R. China; 3 Institute of High Performance Computing (IHPC), A*STAR (Agency for Science, Technology and Research), 1 Fusionopolis Way, Connexis #16-16, Singapore 138632, Republic of Singapore; 4 Department of Chemistry, National University of Singapore, 3 Science Drive 3, Singapore 117543, Republic of Singapore

## Abstract

Lithium nitrate (LiNO_3_) is known as an important electrolyte additive in lithium-sulfur (Li-S) batteries. The prevailing understanding is that LiNO_3_ reacts with metallic lithium anode to form a passivation layer which suppresses redox shuttles of lithium polysulfides, enabling good rechargeability of Li-S batteries. However, this view is seeing more challenges in the recent studies, and above all, the inability of inhibiting polysulfide reduction on Li anode. A closely related issue is the progressive reduction of LiNO_3_ on Li anode which elevates internal resistance of the cell and compromises its cycling stability. Herein, we systematically investigated the function of LiNO_3_ in redox-shuttle suppression, and propose the suppression as a result of catalyzed oxidation of polysulfides to sulfur by nitrate anions on or in the proximity of the electrode surface upon cell charging. This hypothesis is supported by both density functional theory calculations and the nitrate anions-suppressed self-discharge rate in Li-S cells. The catalytic mechanism is further validated by the use of ruthenium oxide (RuO_2_, a good oxygen evolution catalyst) on cathode, which equips the LiNO_3_-free cell with higher capacity and improved capacity retention over 400 cycles.

With high energy density and low cost, lithium-sulfur (Li-S) batteries are considered among the most promising candidates for next-generation energy storage devices^1^,^2^,^3^. The history of Li-S battery research dates back to 1960s^4^, with most of the early works (1960–1990s) in primary batteries focusing on electrolyte optimization and sulfur utilization improvement^5^. The early research on the rechargeable ones, however, encountered the serious issue of fast capacity decay with very limited number of cycles (<20)^6^,^7^,^8^,^9^. The main cause of the poor rechargeability has been known as lithium polysulfide redox shuttles (LiPSs, soluble intermediates in charge/discharge reactions), which internally short-circuit the battery and display an ultra-long voltage plateau at ~2.35 V on charging^10^. Tricks were employed to rectify the cycling behavior by fixing the charge time in each subsequent cycle, which however led to partial charging with little improvement to the fast capacity decay of the batteries^6^. In addition, LiPSs redox shuttle was also found responsible for high self-discharge rate, warranting the urgency of research need in the development of high-performance Li-S batteries^11^.

In 2008, Mikhaylik revealed the efficacy of LiNO_3_ in suppressing LiPSs redox shuttles as a big breakthrough, enabling a real rechargeable Li-S battery with a coulombic efficiency (CE) of close to 100%^12^. Coincidentally, the introduction of LiNO_3_ was found to concurrently improve the sulfur utilization rate in the cathode^13^. These promising results have made LiNO_3_ the most important electrolyte additive and almost appear in every reported Li-S cell thereafter^14^,^15^,^16^,^17^,^18^. The function of LiNO_3_, according to the prevailing understanding, is to form an electrically insulating layer on Li anode via a spontaneous reduction reaction which prevents further reduction of polysulfides thereon^19^. The passivation layer was identified as Li_x_NO_y_ by Aurbach *et al.* using Fourier transform infrared (FTIR) spectroscopy and X-ray photoelectron spectroscopy (XPS)^13^. Later, Wen and co-workers reported LiNO_3_-induced formation of smooth and dense solid electrolyte interphase (SEI) layer on Li anode^20^, serving as a protection layer to the reductive Li metal^21^. Inspired by these promising results, significantly more efforts were devoted to rechargeable Li-S battery research on nanostructured sulfur cathode development, leading to the revelation of a large number of interesting materials and findings^22^,^23^,^24^,^25^,^26^. Nevertheless, recent work of K. Amine’s group showed the irrelevance of redox shuttle suppression to the Li_x_NO_y_ layer^27^, triggering new thinking on future Li-S battery development. Another related phenomenon that echoes this argument is the finding of progressive LiNO_3_ reduction on Li anode, which suggests the ineffectiveness of a Li_x_NO_y_ layer in prevention of LiPSs reduction^28^. All these unambiguously justified the necessity of relooking into the role of LiNO_3_ in rechargeable Li-S batteries. Herein, we revisit some key factors that influence LiPSs redox shuttles, clarify the roles of Li_x_NO_y_ passivation layers, and propose a new mechanism that redox shuttle suppression in the presence of LiNO_3_ is due to the strong binding between the soluble high-order LiPSs and nitrate (NO_3_^−^) anions adsorbed on carbon substrate, which promotes the oxidation of polysulfide to sulfur on charging. This is supported by density functional theory (DFT) calculations and NO_3_^−^-induced low self-discharge rate in Li-S batteries. As the progressive reduction of LiNO_3_ compromises long-term cycling stability of Li-S batteries by gradual increase of the internal resistance, we propose the substitution of soluble LiNO_3_ with solid oxide catalyst of good polysulfide oxidation reaction (PSOR) activity, to which ruthenium oxide was found as a preferable choice.

## Results and Discussion

### Rationale of experimental conditions

Initial coulombic efficiency (ICE) of a rechargeable battery reflects the reversibility of its cell reactions in the first cycle and is typically above 90% for commercial Li-ion batteries. In a Li-S battery with LiNO_3_-free electrolyte, ICE varies notably due to the different extent of redox shuttles. To clarify the role of LiNO_3_, general factors that influence ICE must be fixed, e.g. Li salts concentration and applied current^29^, in all cells are 1 M LiTFSI in DOL/DME (v/v, 1:1) and 0.1 mA, respectively.

In addition, sulfur loading density (SLD) was found to affect ICE (Fig. S1, Supplementary Information). At SLD <0.8 mg cm^−2^, ICE experienced a quasi-linear decrease with the increase of SLD; whereas at SLD >1.1 mg cm^−2^, increase of SLD elevates ICE slowly. Interestingly, at 0.8 <SLD <1.1 mg cm^−2^ ICE exhibits a buffer-like behavior and fluctuates at 62 ± 5%. This unique feature is ideal for the study of redox shuttle effect, as the effect of SLD to ICE can be neglected if it is fixed at 0.9 ± 0.1 mg cm^−2^.

Moreover, the volume of electrolyte shows a direct impact on ICE (Fig. S2, Supplementary Information). On a Li-S cell with 80 μL of electrolyte, an ultra-long charge voltage plateau at 2.35 V presents with an ICE of 66.4%. In contrast, as the electrolyte was reduced to 10 μL, a similar cell delivers a decent cycling performance with an ICE as high as 99.1%. The notably higher ICE at smaller volume of electrolyte was likely due to the increased viscosity of electrolyte at much higher LiPSs concentration that slowed down the diffusion of LiPSs from cathode to anode. This was supported by the “recovered” low CE when the cell was given sufficient time for relaxation between charges (Fig. S3, Supplementary Information). The electrolyte volume effect to the cycling performance of Li-S cells can be found in a systematic study by Zhang^30^, showing the importance of electrolyte volume optimization to the high performance cells. In this study, we fixed the electrolyte volume as 80 μL in cells for ICE study, and 30 μL in those for cycling/storage tests.

**Figure 1:**
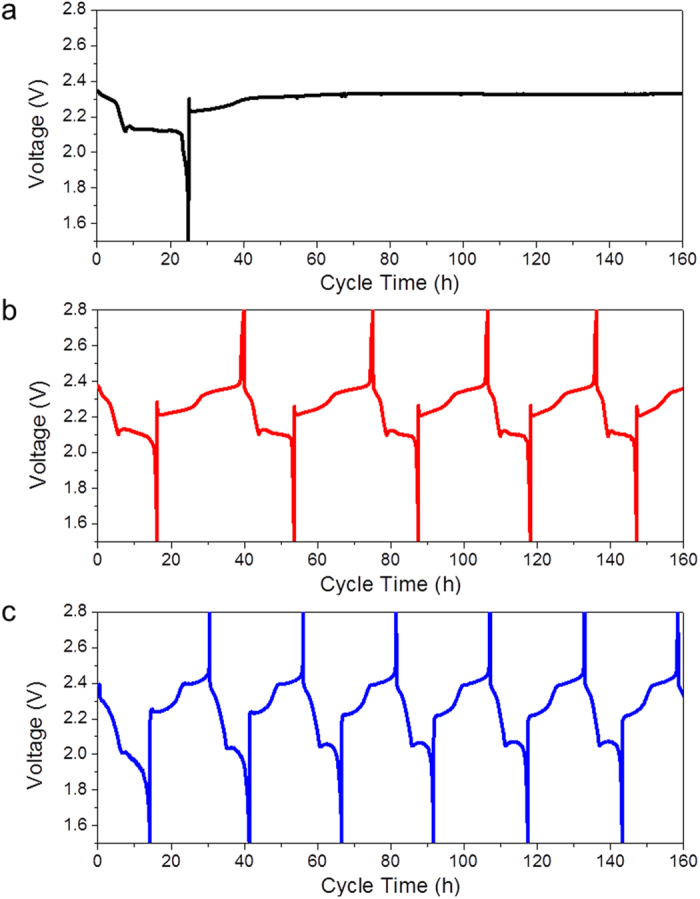
Temperature effect on the galvanostatic discharge-charge voltage profiles of sulfur-graphite composite cycled in LiNO_3_-free electrolyte. The cell was tested at the temperature of 60 °C (**a**), room temperature (**b**) and −10 °C (**c**). At higher temperature the shuttle phenomenon became more distinct.

Further, ICE varies with the change of the operating temperature of the cells (Fig. 1). At a higher temperature, e.g. 60 °C, the severe redox shuttles internally short-circuited the Li-S cell, leading to “infinite” charging; whereas at an operating temperature of −10 °C, ICE of 84.9% was achieved with redox shuttling phenomenon hardly visible. At conventional operating temperature, i.e. room temperature of 25 °C, a Li-S cell with 80 μL electrolyte and SLD of 0.9 mg cm^−2^ delivers ICE of 66.4%. Such cells with moderate ICE values are ideal for the clarification on the role and efficacy of LiNO_3_ additive in Li-S batteries. All experiments were thus conducted at room temperature unless stated otherwise.

### Inability of Li_x_NO_y_ layers on electrodes in redox shuttle suppression

**Figure 2:**
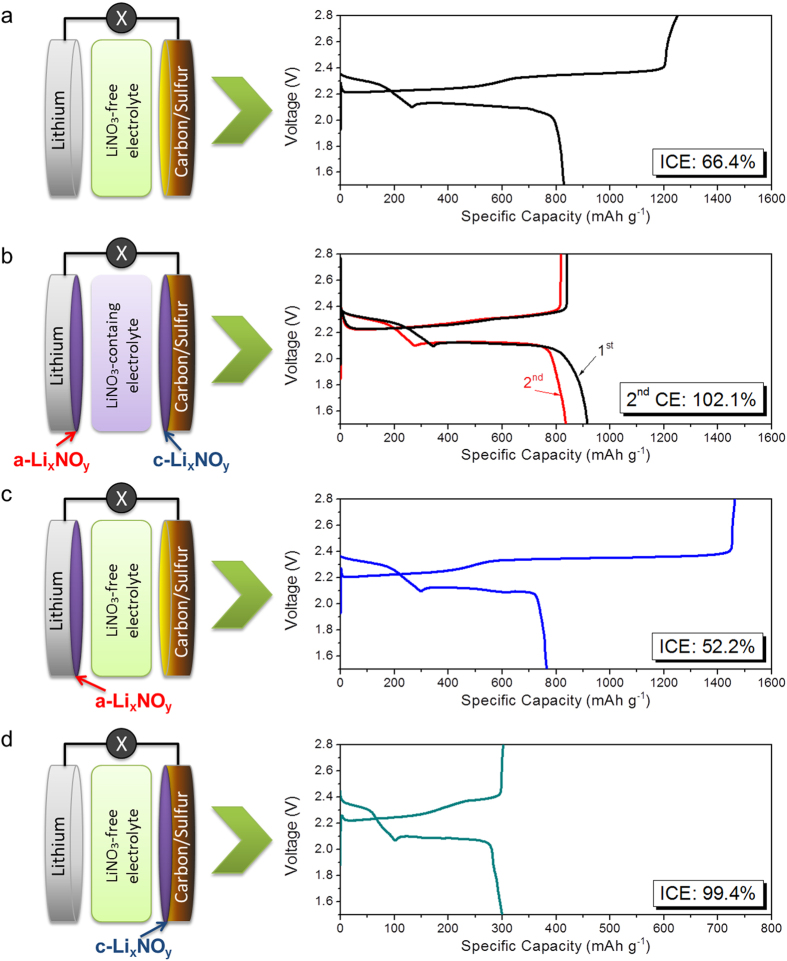
Galvanostatic discharge-charge voltage profiles of Li-S batteries (with sulfur-graphite composite). (**a**) 1^st^ cycle of a Li-S cell cycled in LiNO_3_-free electrolyte, with an ICE of 66.4% as the benchmark. (**b**) The 2 cycles of the cell cycled in LiNO_3_-contained electrolyte which was later dissembled to obtained Li_x_NO_y_ coated Li anode and carbon/sulfur cathode. The reduction of LiNO_3_ on cathode leads to a CE value of ~100%. Li_x_NO_y_ passivation layers formed on both sides of electrodes, assigned to a-Li_x_NO_y_ for anode side and c-Li_x_NO_y_ for cathode side, respectively. (**c**) The first cycle of the reassembled cell from the used Li anode (with a-Li_x_NO_y_ surface coating) and a fresh carbon/sulfur cathode in LiNO_3_-free electrolyte, showing an ICE of 52.2%. (**d**) The first cycle of the reassembled cell with a fresh Li anode and the used carbon/sulfur cathode (with c-Li_x_NO_y_ surface coating) in LiNO_3_-free electrolyte, giving an ICE of 99.4%.

To re-evaluate the efficacy of a Li_x_NO_y_ passivation layer in redox shuttle suppression, we constructed 3 types of Li-S cells, i.e. with Li_x_NO_y_ passivation layer only on anode or cathode, or absent on both electrodes, and get them cycled in LiNO_3_-free electrolyte, respectively. The results show that in the absence of LiNO_3_ and Li_x_NO_y_ passivation layer, a Li-S cell delivers a reproducible initial coulombic efficiency (ICE) of 66.4% (Fig. 2a). At this ICE the LiPSs redox shuttle phenomenon is severe and reflected as an abnormally high charge capacity (or “infinite charging”). In contrast, a replicate cell cycled in LiNO_3_-contained electrolyte gave a reversible efficiency of ~100% in 2^nd^ cycle (Fig. 2b), showing the efficacy of LiNO_3_ in electrolyte for redox-shuttle suppression. The overshoot of ICE above a unit in the 1^st^ cycle (and slightly in the 2^nd^ cycle) was due to the side reaction of LiNO_3_ reduction on cathode (forming Li_x_NO_y_ layer) at the voltage below 1.85 V (Fig. S4, Supplementary Information).

As a “used” Li-S cell with Li_x_NO_y_ layers on both electrodes was disassembled at full-charge state (2.8 V) in an Ar-filled glove box, *a*-Li_x_NO_y_ protected anode and *c*-Li_x_NO_y_ covered cathode were collected (*a* and *c* here stand for anode and cathode, respectively). After cleansed using fresh electrolyte solvent, the *a*-Li_x_NO_y_/Li anode was coupled with a new carbon/sulfur cathode to form a new Li-S cell in LiNO_3_-free electrolyte (Fig. 2c). Surprisingly, with a discharge capacity of 765 mAh g^−1^ the cell showed a charge capacity of 1464 mAh g^−1^, which corresponds to an ICE of as low as 52.2%. This result suggests the inability of *a*-Li_x_NO_y_ layer in LiPSs redox shuttle suppression, echoing the previous report of Amine *et al.*^27^.

Interestingly, a Li-S cell assembled using the cleansed *c*-Li_x_NO_y_ covered cathode and a fresh Li anode in LiNO_3_-free electrolyte was able to deliver an ICE of 99.4% (Fig. 2d), indicating excellent redox-shuttle suppression. Nevertheless, a notable drop in the cell capacity (300 *vs.* 765 mAh g^−1^) is likely due to the dissolution of sulfur/LiPSs into the electrolyte in the previous cell during the preparation of *c*-Li_x_NO_y_ covered cathode. This was supported by a higher capacity retention (511 mAh g^−1^) when the electrolyte volume was reduced from 80 to 30 μL. It is worth noting that this high ICE does not necessarily originate from the *c*-Li_x_NO_y_ layer. The *c*-Li_x_NO_y_ covered cathode with a rough surface was difficult to be fully cleansed, leaving LiNO_3_ residues on (or in the proximity of) the cathode surface or inside the binder polymer matrix which works in its own way to suppress the redox shuttles.

The necessity of *c*-Li_x_NO_y_ for high ICE was further excluded by cycling a freshly assembled Li-S cell of Li anode and carbon/sulfur cathode in LiNO_3_-contained electrolyte with a cut-off voltage of 2.0 V, whereby the reduction of LiNO_3_ on cathode unlikely took place and hence a *c*-Li_x_NO_y_ layer was absent^31^. Nonetheless, the ICE value was found as high as that of the cell discharged to 1.5 V with the formation of *c*-Li_x_NO_y_ (Fig. S5, Supplementary Information). Similar experiment has also been carried out by Zhang recently and NO_3_ radical intermediate was proposed as the key in shuttle suppression^32^. In our experiment, LiNO_3_ was replaced by NaNO_3_ in the electrolyte (Fig. S6, Supplementary Information) and a high ICE was also obtainable. These results have clearly suggested that instead of *c*-Li_x_NO_y_, it was NO_3_^−^ anions adsorbed on (or in the proximity of) cathode suppressing the redox shuttles.

### Stronger binding of LiPSs to NO_3_^−^-theoretical calculations and self-discharge test

**Figure 3:**
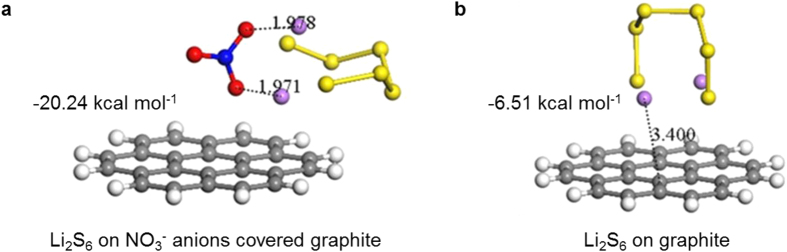
Optimized structures of Li_2_S_6_ on NO_3_ covered graphite and pure graphite models, and the corresponding adsorption energies. (**a**) Li_2_S_6_ on NO_3_^−^ covered graphite; (**b**) Li_2_S_6_ on graphite. Red, blue, yellow, purple, grey and white balls represent O, N, S, Li, C and H atoms, respectively. The numbers indicate the bond lengths in Å.

A possible mechanism for redox shuttle suppression by NO_3_^−^ anions on (or in the proximity of) the graphitic electrode surface is that these anions promote/catalyse a faster oxidation of soluble high-order LiPSs into element sulfur on charging such that fewer LiPSs diffuse to anode surface to undergo a reduction reaction. At higher charge voltage (2.35 V plateau) the redox shuttle phenomenon is a competing process with the oxidation of Li_2_S_4_ → Li_2_S_6_ → Li_2_S_8_ → S_8_. With Li_2_S_6_ as the most stable LiPS^33^, a stronger binding of Li_2_S_6_ to NO_3_^−^ anions on (or in the proximity of) graphite than directly to C atoms of graphite would support the proposed oxidation mechanism^34^. Hence, we calculated the adsorption energies of Li_2_S_6_ onto NO_3_^−^ anions covered graphite and onto pure graphite in electrolyte solvent, respectively. The density functional theory (DFT) modelling data (Fig. 3) shows that on NO_3_^−^ anions covered graphite Li atoms in Li_2_S_6_ “bond” to O atoms in NO_3_^−^ with a “bond-length” of 1.978 or 1.971 Å, comparable to a typical N−H hydrogen bond (1.97 Å). In contrast, on pure graphite Li atoms interacted to C atoms. The Li−C distance in this case was 3.400 Å, which came to the edge of distance for dispersive interaction. Clearly, it has suggested much stronger Li−O interactions between Li and O of NO_3_^−^ than Li−C interactions between Li and C of graphite. This is further supported by the larger adsorption energy for Li_2_S_6_ on NO_3_^−^ (−20.24 kcal mol^−1^) than that on pure graphite (−6.51 kcal mol^−1^). Similar scenario has also been reported previously by Wang *et al.* in a study of graphene oxides^35^. Such notably strong binding can be seen as the first step of the proposed catalytic mechanism. Since it was known that LiPSs→sulfur conversion on pure carbon was hardly possible in the absence of LiNO_3_^36^,^37^, the strong LiPSs/NO_3_^−^ bonding enabled oxidation conversion of polysulfides on cathode upon charging of the battery could be seen as a catalytic process.

**Figure 4:**
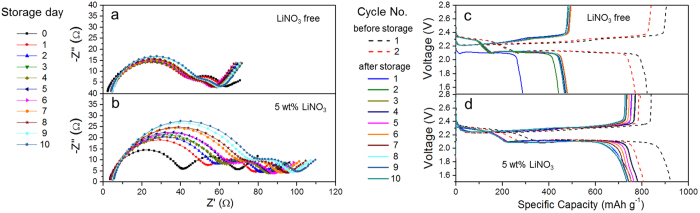
Self-discharge test of Li-S batteries (with sulfur-graphite composite). Nyquist plots and capacity change of the cells with no LiNO_3_ (**a,c**), 5.0 wt% of LiNO_3_ (**b,d**) stored at room temperature over 10 days. Electrolyte volume: 30 μL.

The strong LiPSs/NO_3_^−^ interaction should also help suppress the reduction of S_8_ to Li_2_S_6_ for storage, which is reflected as lower self-discharge rate in battery. This was proven by monitoring the impedance changes of a fully-charged Li-S cell with electrolyte containing 5.0 wt% of LiNO_3_ over 10 days of storage at room temperature, using its LiNO_3_-free counterpart as the control (Fig. 4a,b). In the Nyquist plot the first semi-circle at high frequency range was attributed to the resistance of solid electrolyte interface (R_SEI_), whereas the second one at middle frequency range was ascribed to the charge transfer (R_ct_). The simulation data shows that for the cell with LiNO_3_-contained electrolyte R_SEI_ increased from 37.2 to 70.1 Ω over 10 days (Fig. S7, Supplementary Information), which was notably higher than that of its LiNO_3_-free counterpart (from 38.8 to 42.3 Ω). Fast increase of R_SEI_ in the presence of LiNO_3_ electrolyte additive is attributed to progressive reduction of LiNO_3_ on Li anode^29^. Here, the drop of R_ct_ is more of a concern, as it reflects the state-of-charge of a cell^31^. R_ct_ is larger for higher charged state, and its sharper decrease indicates faster self-discharge. Thus, the slower drop of R_ct_ (27.1 → 24.0 Ω, or 3.1 Ω) for the cell with LiNO_3_-contained electrolyte as compared to that of its LiNO_3_-free counterpart (22.8 → 12.8 Ω, or 10.0 Ω) is suggesting the clear inhibition of self-discharge by LiNO_3_. This is understandable, as NO_3_^−^ anions covered graphite were able to hold LiPSs more tightly and thus notably slowed down the self-discharge process (S_8_ → LiPSs).

The inhibited self-discharge in cells with LiNO_3_-contained electrolyte was echoed by its higher open circuit voltage (OCV, 2.33 V) after 10 days of aging at room temperature as compared to that of the LiNO_3_-free counterpart (2.16 V, Fig. S8, Supplementary Information). The self-discharge suppression is achieved through the strong interactions between Li and O of NO_3_^−^ anions covered graphite and irrelevant to the presence of the *c*-Li_x_NO_y_ layer on cathode, evidenced by a similar OCV when setting a higher cut-off voltage (2.0 V). In cells with LiNO_3_-free electrolyte, self-discharge over time led to the disappearance of the upper voltage plateau corresponding to the series reductions of S_8_ → Li_2_S_8_ → Li_2_S_6_ → Li_2_S_4_ in the following discharge (Fig. 4c). As LiPSs in electrolyte can diffuse to the surface of Li anode and eventually precipitate thereon in the form of solid Li_2_S/Li_2_S_2_^13^, irreversible capacity decay was seen with only 61.5% of capacity retention after the storage of 10 days (475 *vs.* 772 mAh g^−1^). In sharp contrast, cells with LiNO_3_-contained electrolyte were able to retain 97.0% of capacity (783 *vs.* 807 mAh g^−1^) over the same period (Fig. 4d). The significantly suppressed self-discharge in the presence of LiNO_3_ provides a solid support to the stronger interaction of NO_3_^−^ anions on graphite to LiPSs (Li_2_S_6_) in Li−O “bonding” mode. This further supported the catalytic mechanism of redox-shuttle suppression by LiNO_3_.

Despite its high efficacy on LiPSs redox shuttle suppression and self-discharge mitigation, use of LiNO_3_ in electrolyte brings in two noticeable drawbacks to the cycling stability of Li-S cells. One is the progressive reduction of LiNO_3_ on Li anode, elevating cell internal resistance which lowers the discharge voltage^28^; the other is the formation of Li_x_NO_y_ passivation layer on cathode at low voltage which destabilizes the cathode upon charging and induces faster capacity decay^31^,^38^. It is worth noting that cells with LiNO_3_ in electrolyte are generally in a non-equilibrium state, whereby the free LiNO_3_ will be gradually depleted by continuous reduction on both electrodes. Eventually NO_3_^−^ anions on (or in the proximity of) graphite will diffuse into the electrolyte and get consumed as well. This is evidenced by the clear decrease of CE in long-term cycling test of cells using LiNO_3_-contained electrolyte in previous studies^16^,^39^,^40^,^41^. Searching of a replacement for LiNO_3_ becomes necessary for Li-S batteries with long-term cycling stability.

### Replacing LiNO_3_ with transition metal oxides

**Figure 5:**
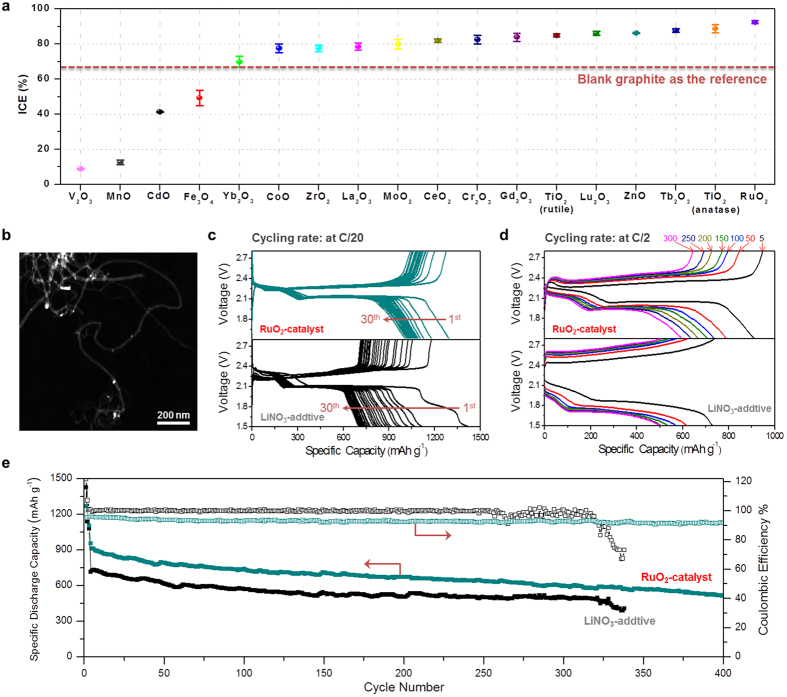
(**a**) ICE data of the Li-S cells with the introduction of different transition metal oxides on graphite substrate. (**b**) HAADF-STEM image of RuO_2_-MWCNTs composite. (**c,d**) Galvanostatic discharge-charge voltage profiles of the cell with RuO_2_ catalyst cycled in LiNO_3_-free electrolyte (top) and the bared MWCNTs-sulfur electrode cycled in the electrolyte with 2.0 wt% of LiNO_3_ additive (bottom) at the cycling rate of C/20 (from 1^st^ to 30^th^ cycle) and C/2 (in 5^th^, 50^th^, 100^th^, 150^th^, 200^th^, 250^th^ and 300^th^ cycle). (**e**) Comparison of the cycling performance of the cell with RuO_2_ catalyst and that with LiNO_3_ additive (at C/2). The cells were first stabilized at C/20 for 3 times.

Different from Zhang’s proposal of NO_3_ radical intermediate as the key to promote LiPSs→sulfur conversion^32^, we believe that the shuttle suppression could be achieved by incorporating solid transition metal oxides into cathode as a catalyst of polysulfide oxidation reactions (PSOR). Some transition metal oxides were employed previously as polysulfide absorbents to improve the performance of Li-S cells, with Magnéli-phase Ti_4_O_7_ being a successful example^42^,^43^. Moreover, MnO_2_^44^, ZrO_2_^45^, La_2_O_3_^46^, NiFe_2_O_4_^47^ and Ni_3_(NO_3_)_2_(OH)_4_^48^ as a major component in cathode, were found to improve the Li-S cell performances in different ways. The new understanding on the role of LiNO_3_ in this work makes us believe that transition metal oxides may be used in low content to “catalyze” the oxidation of the adsorbed polysulfide, if their interactions are strong. To validate this catalytic mechanism, we introduced low content (<3 wt% in the sulfur electrode) of transition metal oxides (18 in total) to investigate their activities towards PSOR. In each experiment one of the 18 transition metal oxides, selected from Period 4 (Fig. S9, Supplementary Information), 5 (Fig. S10, Supplementary Information), and rare earth elements in the lanthanide series (Fig. S11, Supplementary Information) of the periodic table, was loaded onto graphite. The composition and structure of these graphite/oxide composites were confirmed by their respective X-ray diffraction (XRD) patterns (Fig. S12, Supplementary Information). After mixing with sulfur to form the cathode, the shuttle effect of LiPSs in the obtained Li-S cell was studied. The ICE data (Fig. 5a) clearly shows that V_2_O_3_, MnO, CdO and Fe_3_O_4_ imposed a negative effect on ICE (<50%), whereas anatase-TiO_2_, rutile-TiO_2_, Cr_2_O_3_, ZnO, RuO_2_ and most rare earth oxides improved the ICE (>80%) as compared to bare graphite (ICE: 66.4%). It is noteworthy that a direct correlation between CE value and different transition metal oxide here would be hardly achievable. Their binding affinity to polysulfides could be an important factor which, however, is extremely difficult to be accurately quantified due to various complications, e.g. crystal phase, dominating surface atoms, orientation, overall exposure to surface of electrode made from slurry method, etc. These factors could vary notably for different metal oxide species. One point to note could be the adverse impact of V_2_O_3_ and MnO with notable reduction power, which may compete with the “polysulfide oxidation reaction” and make the latter more difficult to take place. A clearer picture may be available after more discrete research on various transition metal oxides are studies in great details in future.

Coincidentally, RuO_2_, an efficient catalyst towards oxygen evolution reaction (OER)^49^, was found to be the most effective catalyst towards PSOR among these oxides, furnishing Li-S cells with the highest CE (92.5%) and superior cycling stability as compared to their LiNO_3_-based counterparts (Fig. 5b–d). The discharge capacity can be improved by replacing graphite (17.8 m^2^ g^−1^, Fig. S13, Supplementary Information) with large surface area carbon, e.g. multi-walled carbon nanotubes (MWCNTs, 256.3 m^2^ g^−1^). Figure 5b shows the high-angle annular dark-field scanning transmission electron microscopy (HAADF-STEM) image of MWCNTs decorated with RuO_2_ and the bright dots indicate the location of well-dispersed RuO_2_ nanoparticles. By employing a cathode of MWCNTs/sulfur composite with 2.87 wt% RuO_2_, initial discharge capacity of as high as 1298 mAh g^−1^ was achieved at a current density of C/20 (1C = 1672 mAh g^−1^, Fig. 5c). In contrast, the MWCNTs/sulfur composite based Li-S cells without RuO_2_ gave additional two potential plateaus at lower voltages (<1.89 V, Fig. S14, Supplementary Information), attributing to the reduction of LiNO_3_ which is detrimental to the long-term cycling stability of the cell. One may eliminate LiNO_3_ reduction at cathode side by setting a higher cut-off voltage, as suggested by Aurbach^38^; however, the progressive LiNO_3_ reduction on Li anode remains an issue^28^. It is hence not surprising to see faster capacity fading in cells cycled in LiNO_3_-contained electrolyte than in the ones with RuO_2_ as a replacement (Figs 5c and S15, Supplementary Information). The improvement of battery performance becomes more pronounced at elevated cycling rate (C/2), reflected as higher capacity and smaller voltage gap (Fig. 5d). For instance, in 5^th^ cycle the cell with RuO_2_ delivers a discharge capacity of 912.4 mAh g^−1^, which is notably higher than the one with LiNO_3_ additive (729.8 mAh g^−1^). Meanwhile, the much smaller voltage gap (0.266 *vs.* 0.686 V, in 5^th^ cycle at a discharge capacity of 400 mAh g^−1^) corresponds to a higher round-trip efficiency of the cell equipped with RuO_2_ (84.1 *vs.* 61.6%). The lower discharge voltage at higher rate for battery using LiNO_3_ additive was likely due to the higher impedance caused by the introduction of Li_x_NO_y_ layer as a result of LiNO_3_ reduction. This again shows the advantage of using transition metal oxide, whereby the Li_x_NO_y_ layer formation can be avoided. In a long-cycling test for batteries with transition metal oxide or LiNO_3_, similar capacity fading trends were observed for both cells in the first 250 cycles except higher capacity for the former in the same cycle. The similar fading trend was likely due to the loss (with cycling) of active materials (sulfur) on cathode which still remains an issue to be solved. The lower capacity (retention) at C/2 for the one with LiNO_3_ additive was due to its higher impedance from the Li_x_NO_y_ layers as we discussed before. Adverse effect rising from the LiNO_3_ progressive reduction started to become more prominent from 250 cycles, and the cells become unstable as clearly evidenced by the oscillated CE values (Fig. 5e). The same phenomenon is also observed in Li-S cells with graphite/sulfur electrodes (Fig. S16, Supplementary Information) and also a recent study by Janek *et al.*^50^. Interestingly, a Li-S cell with RuO_2_ on cathode exhibited a constant CE and was able to deliver a discharge capacity of 513.3 mAh g^−1^ (@C/2) even after 400 cycles.

## Conclusion

In summary, we re-examined the efficacy of Li_x_NO_y_ layers on both anode and cathode in redox shuttle suppression, and found both ineffective. Previously observed convincing phenomenon of redox shuttle suppression by LiNO_3_ additive in electrolyte could have functioned through the NO_3_^−^ anions on or in the proximity of the graphitic surface in sulfur cathode. These anions bind strongly to LiPSs via “bond-like” interaction, facilitating fast LiPSs→sulfur conversion which mitigates the diffusion of LiPSs to anode in charge process. The stronger binding was confirmed by DFT calculations and echoed by LiNO_3_-enabled low self-discharge rate in Li-S batteries. To replace LiNO_3_ for long-term cycling stability, low content insoluble solid transition metal oxide was incorporated into carbon substrate in sulfur cathode. RuO_2_, a prominent OER catalyst, enables an ICE of 92.5% with improved long-term cycling stability. This work opens a new research path for the development of Li-S batteries, i.e. using polysulfide oxidation catalysts as enablers to realize long-term cycling stability.

## Methods

### Materials and electrochemical characterizations

Primary synthetic graphite (TIMCAL TIMREX® KS6), multi-walled carbon nanotubes (MWCNTs, Aldrich, O.D. × L: 6–9 nm × 5 μm, purity: >95%), precipitated sulfur powder (Alfa Aesar, 99.5%) and poly(vinylidenefluoride) (PVDF, Solef® 5130) were used as received. The graphite/sulfur cathode was prepared by manually mixing graphite and sulfur (w/w, 1:1) in an agate mortar to form a graphite/sulfur composite which was then formulated to a slurry with PVDF in 1-methyl-2-pyrrolidinone (NMP, anhydrous, Aldrich) (w/w: 95/5), and cast evenly onto a piece of aluminum (Al) foil. For the CNT/sulfur cathode, PVDF content was increased to 10 wt% to ensure strong adhesion of active material on Al foil. After drying in air at 80 °C for 2 h, electrode discs of 15 mm in diameter were punched out and transferred into an argon-filled glove box (MBraun) for assembly of Li-S cells. The electrolyte used was 1 M lithium bis(trifluoro-methanesulfonyl) imide (LiTFSI, Aldrich, 99.0%) in a solvent of 1,2-Dimethoxyethane (DME, Alfa Aesar, 99+%) and 1,3-Dioxolane (DOL, Alfa Aesar, 99.5%) (v/v, 1:1), or with additional 5.0 wt% of lithium nitrate (LiNO_3_, Aldrich, 99.0%) as additive. The lithium salts were further dried at 150 °C for 15 h before transferring into the glove box.

The impregnation of RuO_2_ was via co-precipitation method. 0.26 g of MWCNTs (or KS6 graphite) was first dispersed in a solvent (100 mL) of 2-propanol and acetone (v/v, 1:1) under sonication. Then ruthenium chloride (RuCl_3_·xH_2_O, Alfa Aesar) was dissolved in the above suspension. Subsequently, sodium hydroxide solution (0.1 g mol^−1^, 12 mL) was slowly added with vigorous stirring at room temperature. The obtained composite was collected by centrifugation at 6,000 rpm, then thoroughly washed by deionized water until no chloride ions was detected by silver nitrate solution, and finally dried at 180 °C for 15 h. The synthesized ruthenium compound was determined to be rutile RuO_2_ (JCPDS #43-1027) by X-ray diffraction pattern (Bruker D8 Advance) and the loading on carbon is 6.38 wt% (or 2.87 wt% in RuO_2_-MWCNTs-sulfur cathode), confirmed by thermogravimetric analysis (TGA, TA Instruments Q500). TEM image was collected from the monochromated transmission electron microscope (FEI, Titan) in a scanning mode. The RuO_2_-MWCNTs-sulfur cathode is composed of 45 wt% of sulfur, 45 wt% of RuO_2_-MWCNTs composite and 10 wt% of PVDF, with the sulfur loading density of 0.66 mg cm^−2^. The RuO_2_-graphite-sulfur cathode contains 47.5 wt% of sulfur, 47.5 wt% of RuO_2_-graphite composite and 5 wt% of PVDF, with a typical sulfur loading density of ~0.9 mg cm^−2^. The cells were cycled in the electrolyte containing 1 M LiTFSI in DME/DOL (v/v, 1:1), or with additional 2.0 wt% of LiNO_3_ as additive.

Electrochemical performance was studied using CR2032-type coin cells with Li metal (diameter: 15.8 mm) counter electrode and Celgard 2400 separator. The cells were tested on a multichannel battery tester (Shenzhen Neware) at a constant current of 0.1 mA. The cycling voltage range was set between 1.5 and 2.8 V. To clarify the role of Li_x_NO_y_ passivation layers, the Li-S cells were run over 3 cycles and disassembled at full-charge state (2.8 V) in a glove box. The obtained cycled electrodes were washed a few times using a mixture solvent of DME and DOL (v/v, 1:1, pre-treated by metallic lithium stripes over 48 h) before re-assembling into new cells with a fresh counter electrode and LiNO_3_-free electrolyte. Electrochemical impedance spectra (EIS) were recorded on an Autolab instrument in the frequency range of 100 kHz to 50 mHz at room temperature. To amplify the self-discharge effect, the sulfur loading density (SLD) of the cells for storage test was reduced to 0.8 mg cm^−2^.

### Computational methods

A single layer of graphite (coronene structure) was adopted to represent the simplest cluster model of graphite, with Li_2_S_6_ as the most stable LiPSs being used in DFT calculations. The optimized Li_2_S_6_ exhibited a ring-like structure which resembles cyclo-octasulfur (S_8_). The additive NO_3_^−^ and Li_2_S_6_ placed above the plane of the graphite model were used as the starting geometry. Geometry optimization was performed using unrestricted wB97XD density functional in Gaussian 09 suite of program^51^,^52^. The selected functional uses Grimme’s D2 empirical dispersion correction^53^ that accounts for long-range interactions corrections. The 6-31+ G(d,p) basis sets were adopted for H, Li, C, N O and S atoms^54^,^55^,^56^. The selected basis sets contained diffuse functions which describes charged species. Self-consistency-field energy convergence was set at 10^−6^ Hartree. 1,2-dimethoxyethane (DME) is used as the solvent within the polarization continuum model (PCM)^57^. Four parameters are set for this solvent: the static dielectric constant (eps) = 7.2; the molecular radius of the solvent (rsolv) = 2.78255 Å; the density of the solvent (density) = 0.005804 particles Å^−1^ and the molar volume of the solvent (vmol) = 103.7911 cm^−3^. The rest are based on the default parameters of water solvent. Zero-point energy corrections obtained from vibrational frequency calculations in solvent are included in the adsorption energy (*E*_*ad*_) calculations. *E*_*ad*_ is calculated using the formula:


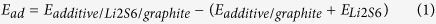


where *E*_*additive/Li2S6/graphite*_, *E*_*additive/graphite*_ and *E*_*Li2S6*_ are the total energy of optimized models of additive/Li_2_S_6_/graphite, additive/graphite, and Li_2_S_6_ polysulfide, respectively. The more negative adsorption energy indicates stronger binding strength.

## Additional Information

**How to cite this article**: Ding, N. *et al.* Building better lithium-sulfur batteries: from LiNO_3_ to solid oxide catalyst. *Sci. Rep.* **6**, 33154; doi: 10.1038/srep33154 (2016).

## Supplementary Material

Supplementary Information
