# Supplementary material for: Building better lithium-sulfur batteries: from LiNO3 to solid oxide catalyst
Source: Sci Rep. 2016 Sep 15;6:33154. doi: 10.1038/srep33154 (PMC5024100; doi:10.1038/srep33154)
Supplement: Supplementary Information [file srep33154-s1.pdf]

Supplementary Information

Building better lithium-sulfur batteries: from $LiNO_3$ to solid oxide catalyst

Ning Ding, Lan Zhou, Changwei Zhou, Dongsheng Geng, Jin Yang, Sheau Wei Chien, Zhaolin Liu, Man-Fai Ng, Aishui Yu, T. S. Andy Hor, Michael B. Sullivan and Yun Zong

Preparation of transition metal oxide-graphite composites

Most of transition metal oxide-graphite composites (5:95, w/w) were prepared via thermal decomposition of nitrate at high temperatures. Metal nitrate was dissolved in the ethanol-water solution (1:1, v/v), and then graphite (KS6) was added and dried under vigorous stirring at 100 °C. For TiO_2, V_2O_3 and MoO_2, the precursors were titanium methoxide ($Ti(OCH_3)_4$), ammonium metavanadate (NH_4VO_3) and molybdic acid ($MoO_3{\cdot}H_2O$), respectively. To fully dissolve $MoO_3{\cdot}H_2O$, ammonium hydroxide solution was gradually added until the solution changed from muddy to clear. The as-prepared powders were collected and finally decomposed at 600 °C for 2 h under Ar atmosphere. The applied low decomposition temperature prevents the carbothermal reaction (usually above 800 °C) between transition metal oxide and graphite. However, at 600 °C only anatase-TiO_2 was obtained and the high-temperature stable rutile-TiO_2 was achieved by heating $Ti(OCH_3)_4$/graphite composite to 900 °C for 1 h. The impregnation of RuO_2 was via co-precipitation method. Ruthenium chloride ($RuCl_3{\cdot}H_2O$) was firstly dissolved in water and stirred for 0.5 h. Then graphite was added into the solution and stirred for 0.5 h at room temperature. Subsequently, sodium hydroxide solution (1.0 M) was slowly added to adjust the pH value to 7. The suspension was continuously stirred for 1 h for aging, then filtered and washed with deionized water several times to remove sodium chloride residual, and finally heated at 150 °C for 15 h.

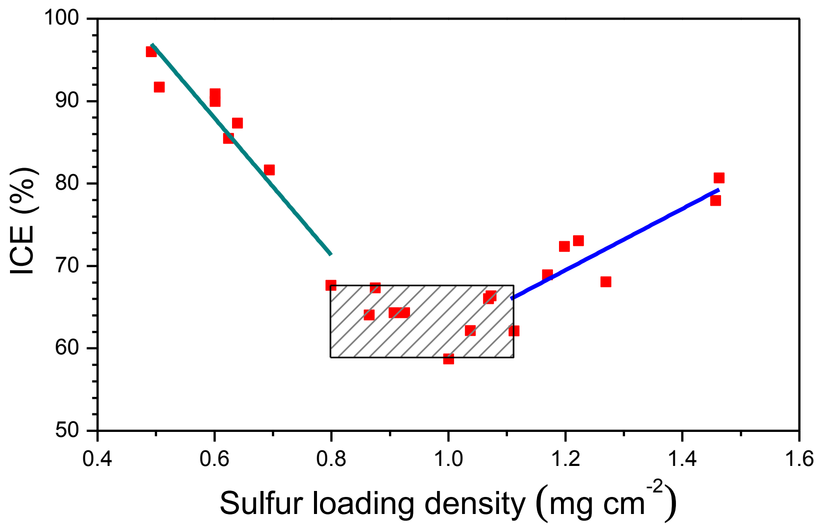

Supplementary Figure S1: Correlation between initial coulombic efficiency (ICE) and sulfur loading density (SLD) of Li-S cells cycled in 80 µL $LiNO_3$-free electrolyte. If SLD < 0.8 mg cm^{-2}, ICE decreased almost linearly with the increase of SLD; whereas for SLD > 1.1 mg cm^{-2}, the increase of SLD led to slightly higher ICE. Interestingly, with 0.8 < SLD < 1.1 mg cm^{-2} ICE falls in the range of 62±5%. This buffer-like behavior allows for the adoption of SLD ~ 0.9±0.1 mg cm^{-2} at which ICE is inert to minor deviation of SLD, facilitating study on additional factors that affect ICE.

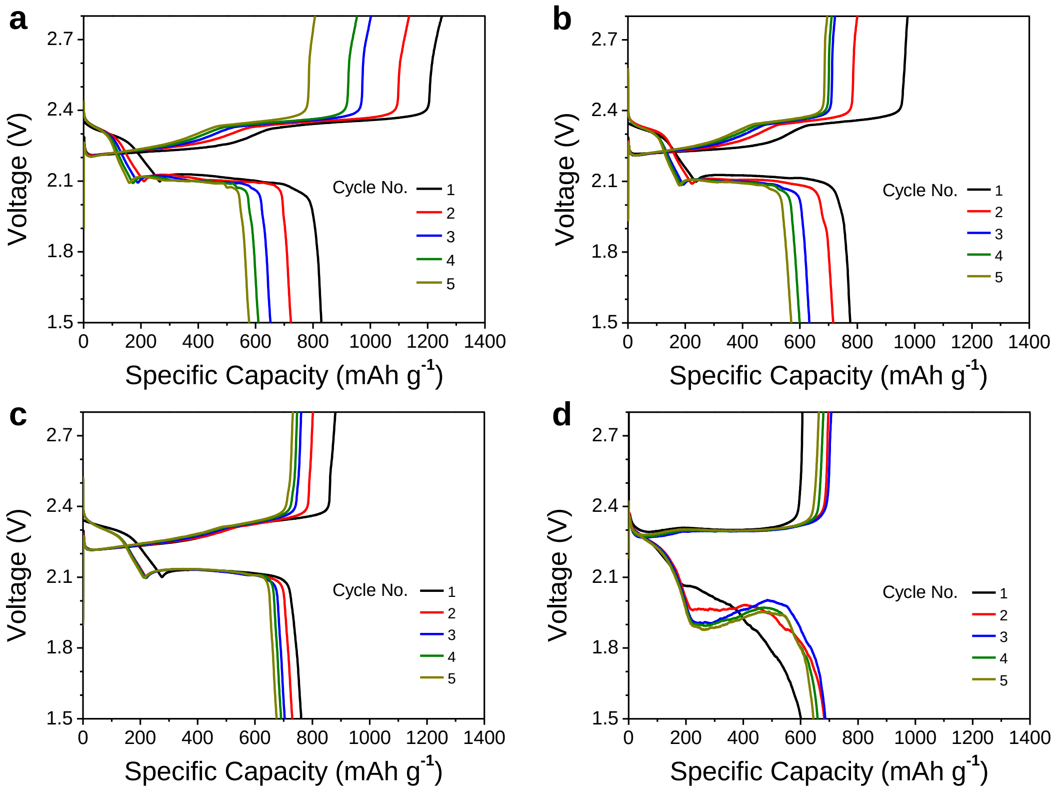

Supplementary Figure S2: Galvanostatic discharge-charge voltage profiles of graphite/sulfur composite cycled in $LiNO_3$-free electrolyte with different volumes: (a) 80 μL (ICE: 66.4%), (b) 60 μL (ICE: 79.4%), (c) 30 μL (ICE: 86.4%) and (d) 10 μL (ICE: 99.1%).

A Li-S cell with 80 μL electrolyte gave an extremely long charge voltage plateau at 2.35 V with an ICE of only 66.4%, indicating severe redox shuttle effect. In contrast, with a reduced volume of electrolyte (e.g. 10 μL) a similar cell exhibited significantly improved cycling performance with ICE as high as 99.1%, showing effective suppression of redox shuttles. The higher ICE at smaller volume of electrolyte was likely due to the increased viscosity at higher LiPSs concentration in electrolyte that slowed down the diffusion of LiPSs from cathode to anode.

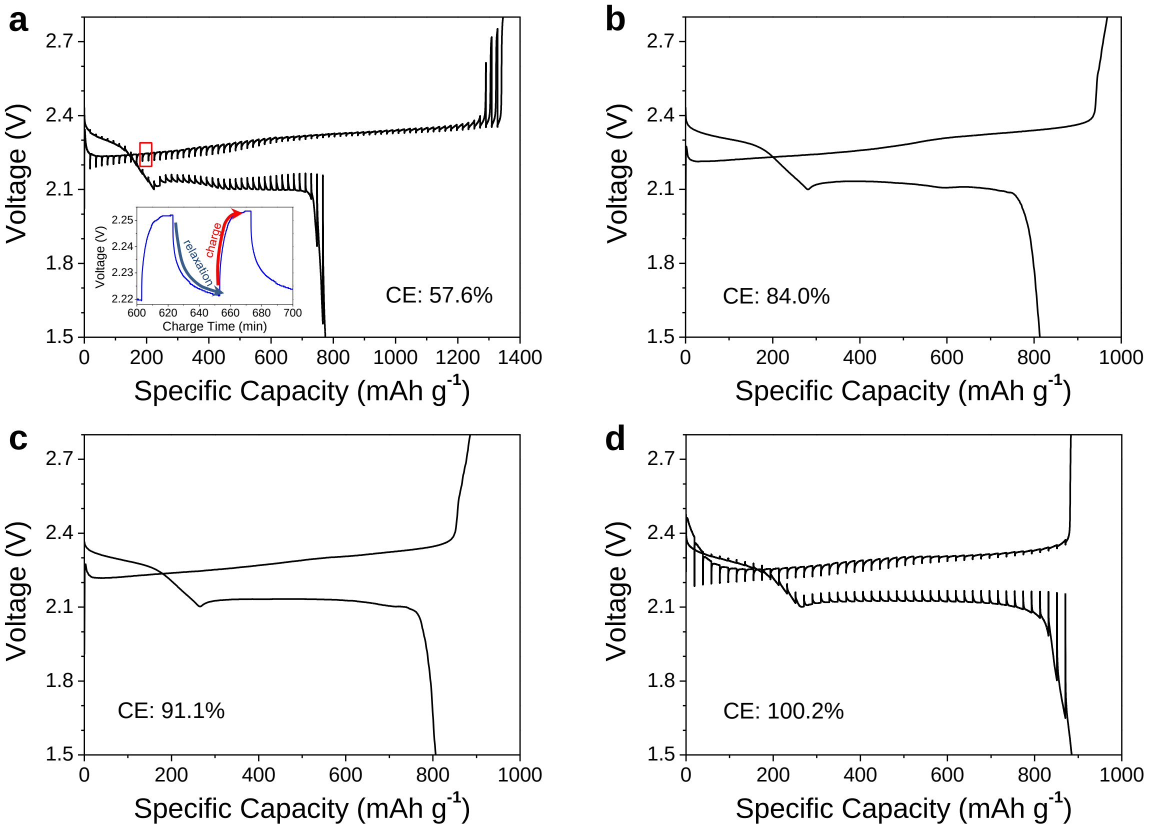

Supplementary Figure S3: Galvanostatic intermittent titration analysis measured by 20 min of galvanostatic charge/discharge (0.1 mA), followed by 30 mins of relaxation time. Cells were cycled in $LiNO_3$-free electrolyte. (a) Galvanostatic intermittent titration curve *vs.* capacity and the inset of galvanostatic intermittent titration curve *vs.* time (two steps marked on charging). Galvanostatic discharge-charge voltage profiles (b) before and (c) after GITT measurement. The relaxation process in GITT test clearly leads to an extremely low CE (57.6%). (d) Galvanostatic intermittent titration curve *vs.* capacity of the cell with $LiNO_3$-contained electrolyte. The addition of $LiNO_3$ can significantly improve CE even in GITT test (still around 100%). Electrolyte volume: 30 μL.

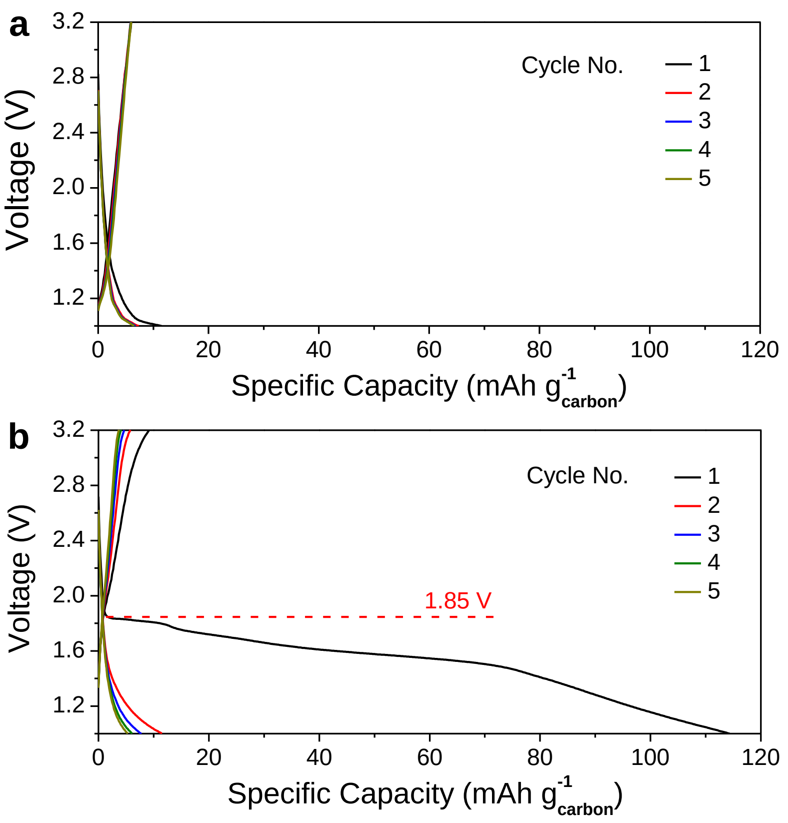

Supplementary Figure S4: Galvanostatic discharge-charge voltage profiles of graphite cycled in (a) $LiNO_3$-free electrolyte and (b) 5.0 wt% $LiNO_3$ contained electrolyte. The reduction of $LiNO_3$ on graphite starts off at the voltage of 1.85 V and the reduction product is irreversible in the following cycles.

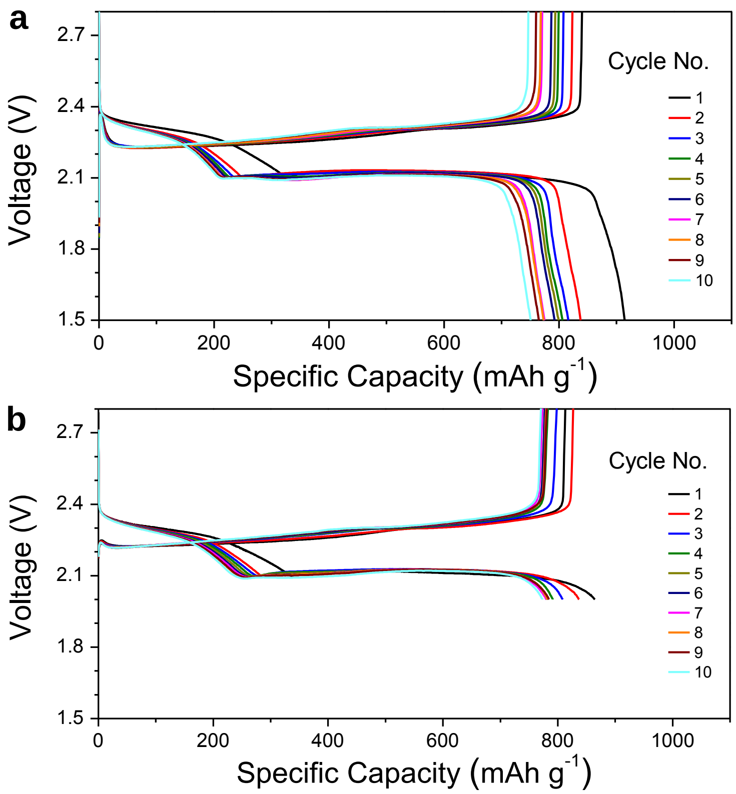

Supplementary Figure S5: Galvanostatic discharge-charge voltage profiles of graphite/sulfur composite cycled in $LiNO_3$-contained electrolyte with different low cut-off voltage. (a) 1.5 V and (b) 2.0 V. The cell with a higher cut-off voltage exhibits better cycling performance.

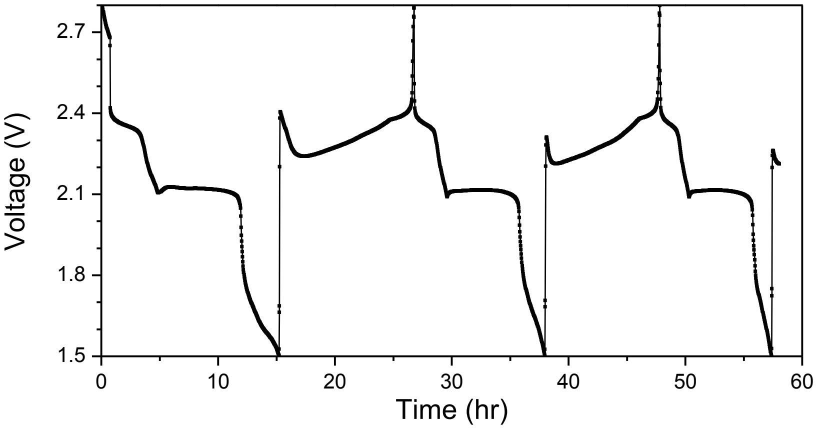

Supplementary Figure S6: Galvanostatic discharge-charge voltage profiles of graphite/sulfur composite with cycled in 5.0 wt% $NaNO_3$-contained electrolyte. The introduction of $NaNO_3$ can also suppress the shuttle phenomenon.

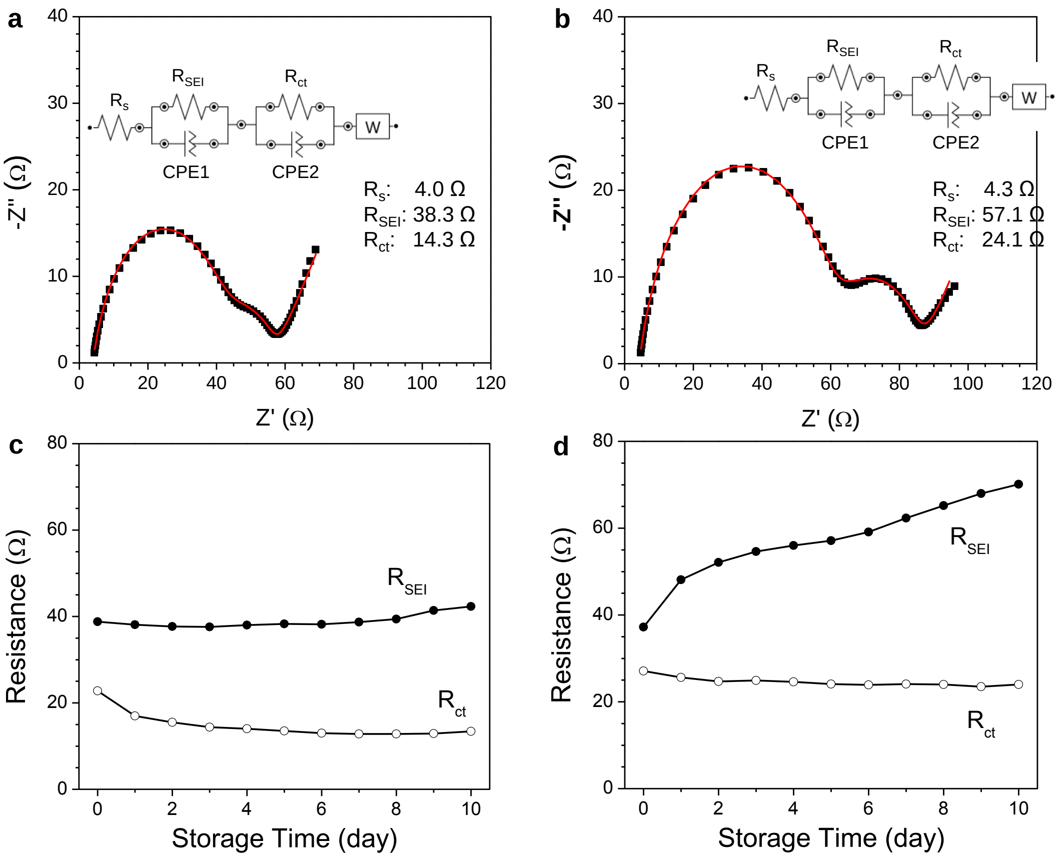

Supplementary Figure S7: Nyquist plots of the cells with (a) $LiNO_3$-free electrolyte and (b) 5.0 wt% of $LiNO_3$ stored at room temperature for 5 days. The inset shows the corresponding equivalent circuit model. Variation of R_{SEI}/R_{ct} *vs.* storage time for the cells with (c) $LiNO_3$-free electrolyte and (d) 5.0 wt% $LiNO_3$ stored at room temperature. Compared with the cell without $LiNO_3$, a faster increase of R_{SEI} is observed for the cell with $LiNO_3$ due to the progressive reaction between $LiNO_3$ and Li anode, whereas R_{ct} remains a constant value in storage, indicating a low self-discharge rate.

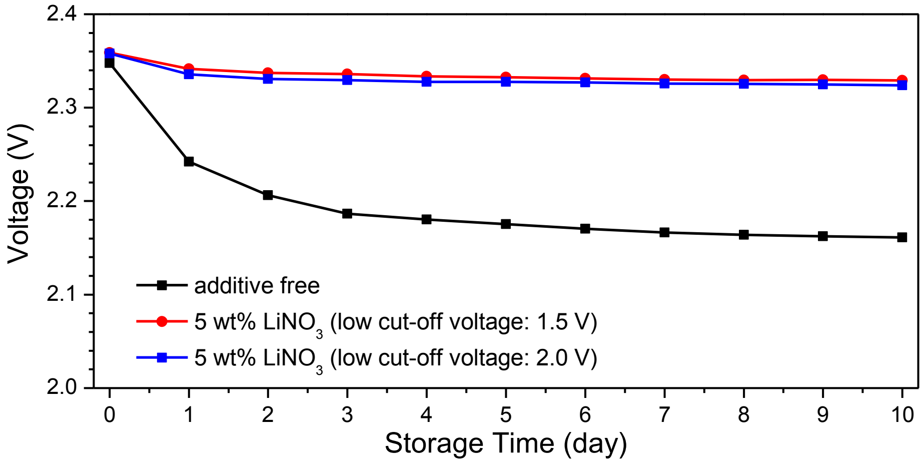

Supplementary Figure S8: Voltage change of the cells with/without $LiNO_3$ stored at room temperature. A similar voltage change of the cells with low cut-off voltages of 1.5 and 2.0 V excludes the influence of Li_xNO_y passivation layer on cathode on self-discharge rate.

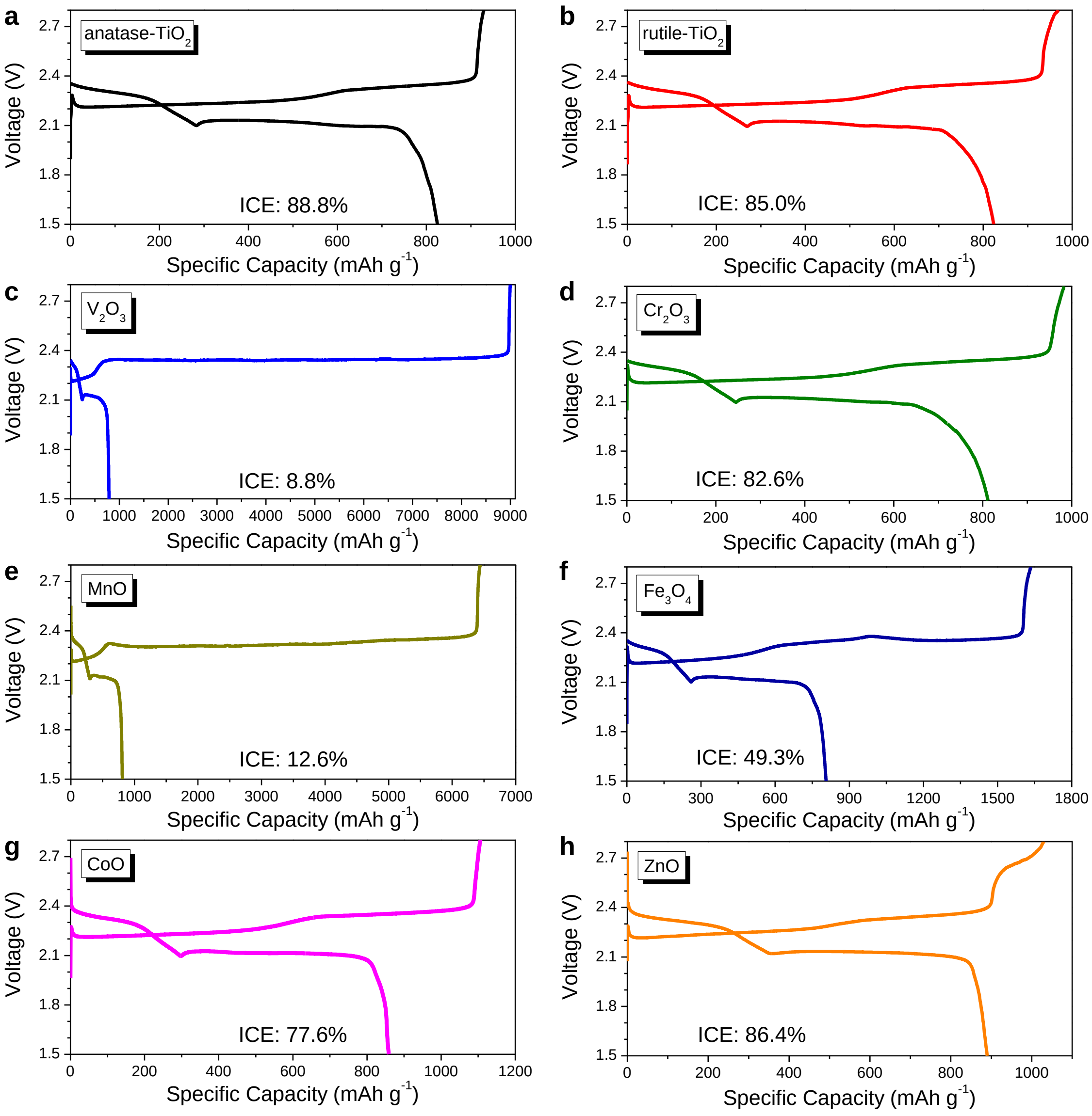

Supplementary Figure S9: Initial discharge-charge voltage profiles of Li-S batteries cycled in $LiNO_3$-free electrolyte with the transition metal oxide (in period 4)-graphite composite (5:95, w/w) as the substrate. (a) anatase-TiO_2, (b) rutile-TiO_2, (c) V_2O_3, (d) Cr_2O_3, (e) MnO, (f) Fe_3O_4, (g) CoO and (h) ZnO. The electrode consists of 50 wt% of sulfur with a loading density is 0.9 mg cm^{-2}. Electrolyte volume: 80 μL. Applied current: 0.1 mA.

With a sulfur loading density of 0.9 mg cm^{-2}, bare graphite can only deliver an ICE of 65% (Figure S1). Among the studied transition metal oxides in period 4, the introduction of V_2O_3, MnO and Fe_3O_4 induces to a lower ICE value, whereas TiO_2 (both phases) and ZnO clearly have a positive effect, with an ICE value of above 85%. The influence of anatase TiO_2 on CE is also approved by Amine et al.[1]

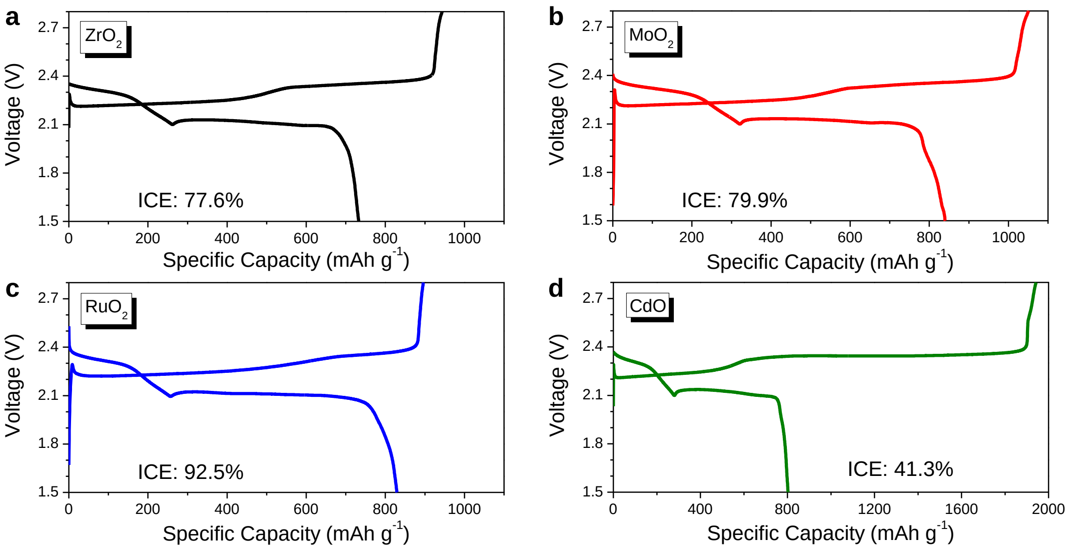

Supplementary Figure S10: Initial discharge-charge voltage profiles of Li-S batteries cycled in $LiNO_3$-free electrolyte with the transition metal oxide (in period 5)-graphite composite (5:95, w/w) as the substrate. (a) ZrO_2, (b) MoO_2, (c) RuO_2 and (d) CdO. The electrode consists of 50 wt% of sulfur with a loading density is 0.9 mg cm^{-2}. Electrolyte volume: 80 μL. Applied current: 0.1 mA.

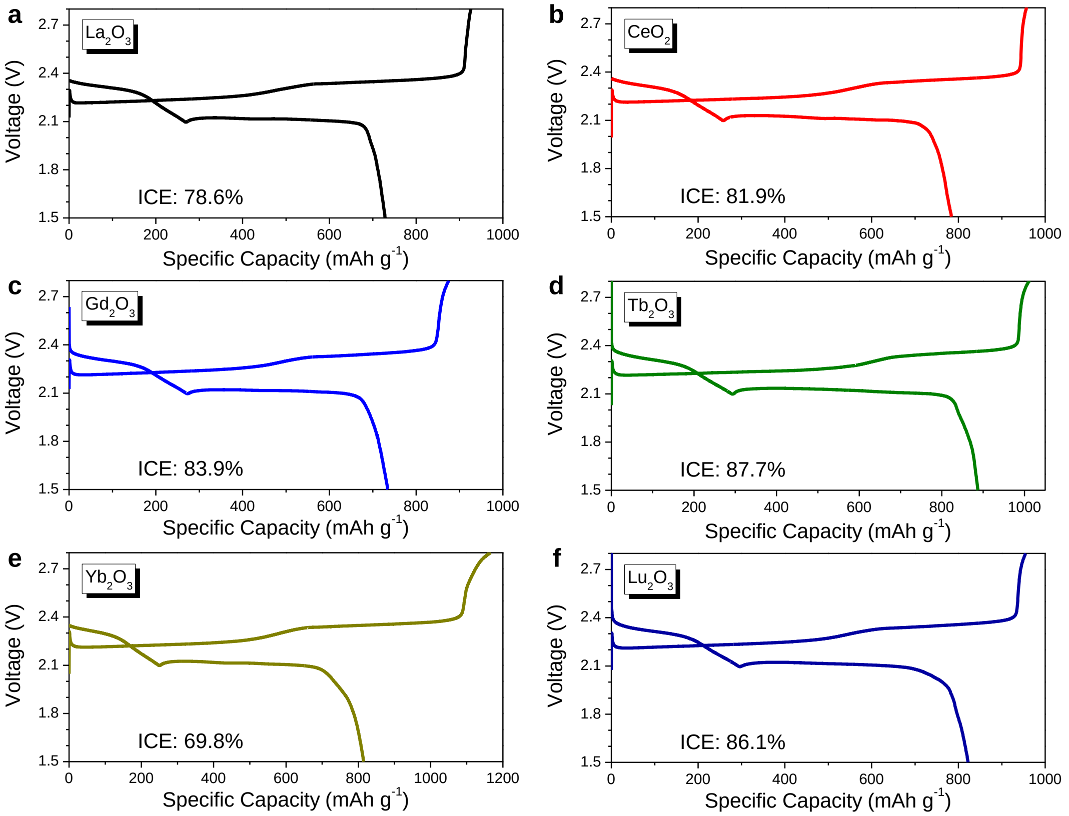

Supplementary Figure S11: Initial discharge-charge voltage profiles of Li-S batteries cycled in $LiNO_3$-free electrolyte with the rare earth oxides in the lanthanide series)-graphite composite (5:95, w/w) as the substrate. (a) La_2O_3, (b) CeO_2, (c) Gd_2O_3, (d) Tb_2O_3, (e) Yb_2O_3 and (f) Lu_2O_3. The electrode consists of 50 wt% of sulfur with a loading density is 0.9 mg cm^{-2}. Electrolyte volume: 80 µL. Applied current: 0.1 mA.

All the studied rare earth oxides can deliver a higher ICE than bare graphite (65%), but are not superior to RuO_2. The introduction of RuO_2 leads to the highest ICE (92.5%) among all 18 transition metal oxides.

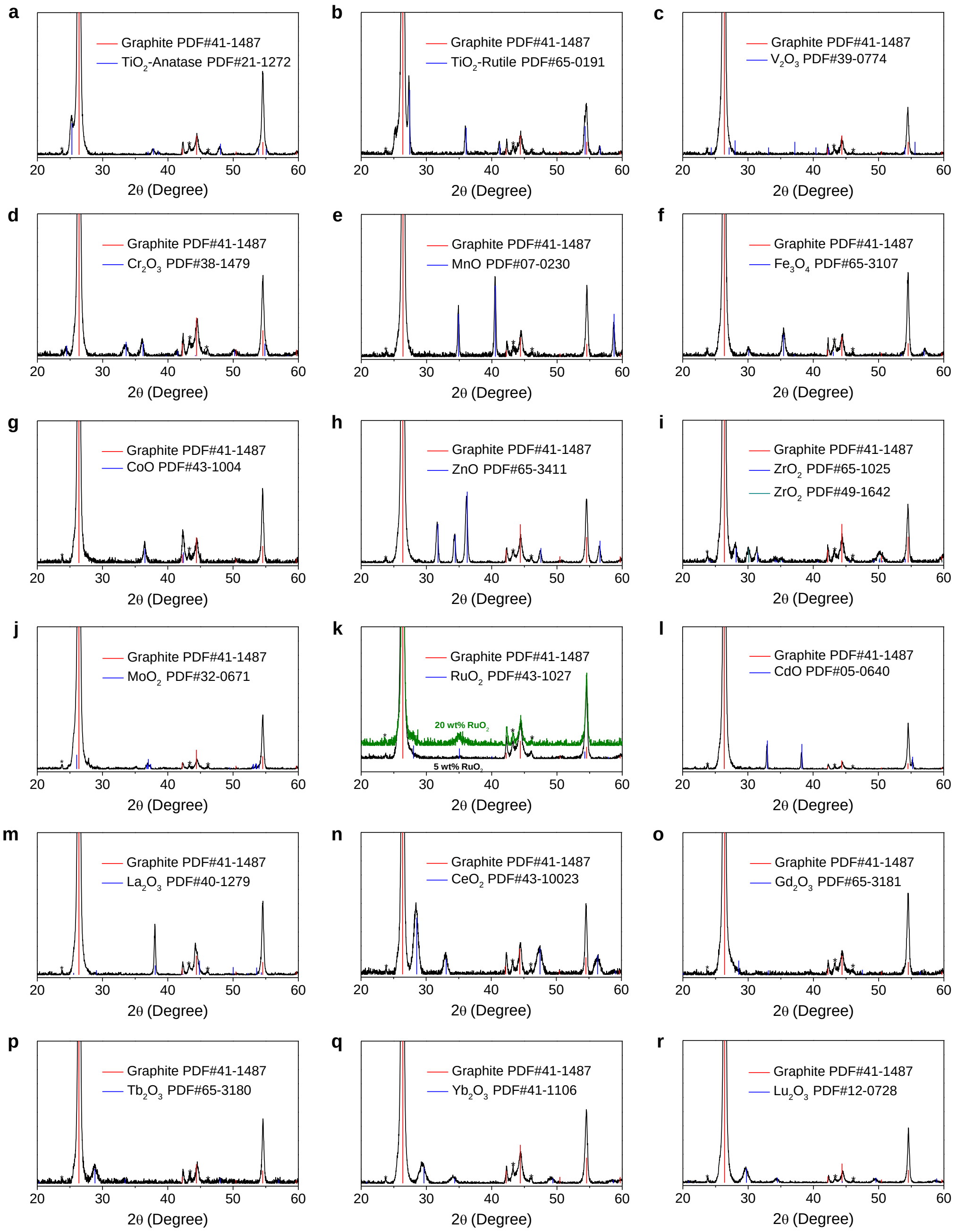

Supplementary Figure S12: XRD patterns of the as-prepared transition metal oxide-graphite composites. (a) pure anatase TiO_2, (b) rutile TiO_2 with trace of anatase TiO_2, (c) amorphous V_2O_3 (the composition is determined by ref. 2), (d) Cr_2O_3 (space group: *R-3c*), (e) MnO (space group: *Fm-3m*), (f) Fe_3O_4 (space group: *Fd-3m*), (g) CoO (space group: *Fm-3m*), (h) ZnO (space group: $P6_3mc$), (i) mixture of monoclinic ZrO_2 (space group: $P2_1/c$) and cubic ZrO_2 (space group: *Fm-3m*), (j) MoO_2 (space group: $P2_1/n$), (k) RuO_2 (space group: $P4_2/mnm$), (l) CdO (space group: *Fm-3m*), (m) La_2O_3 (space group: *P-3m1*), (n) CeO_2 (space group: *Fm-3m*), (o) Gd_2O_3 (space group: *Ia-3*), (p) Tb_2O_3 (space group: *Ia-3*), (q) Yb_2O_3 (space group: *Ia-3*) and (r) Lu_2O_3 (space group: *Ia-3*). The peaks marked with an asterisk arise from the impurity of KS6.

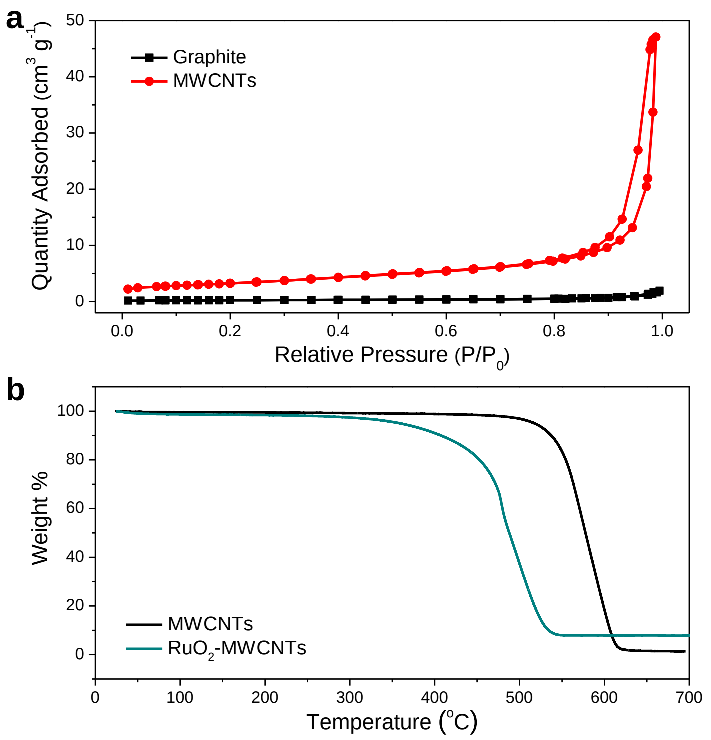

Supplementary Figure S13: (a) Adsorption–desorption isotherms of graphite (BET surface area = 17.8 m^2 g^{-1} and MWCNT (BET surface area = 256.3 m^2 g^{-1}). (b) TGA curves of pure MWCNTs and RuO_2-MWCNTs composite.

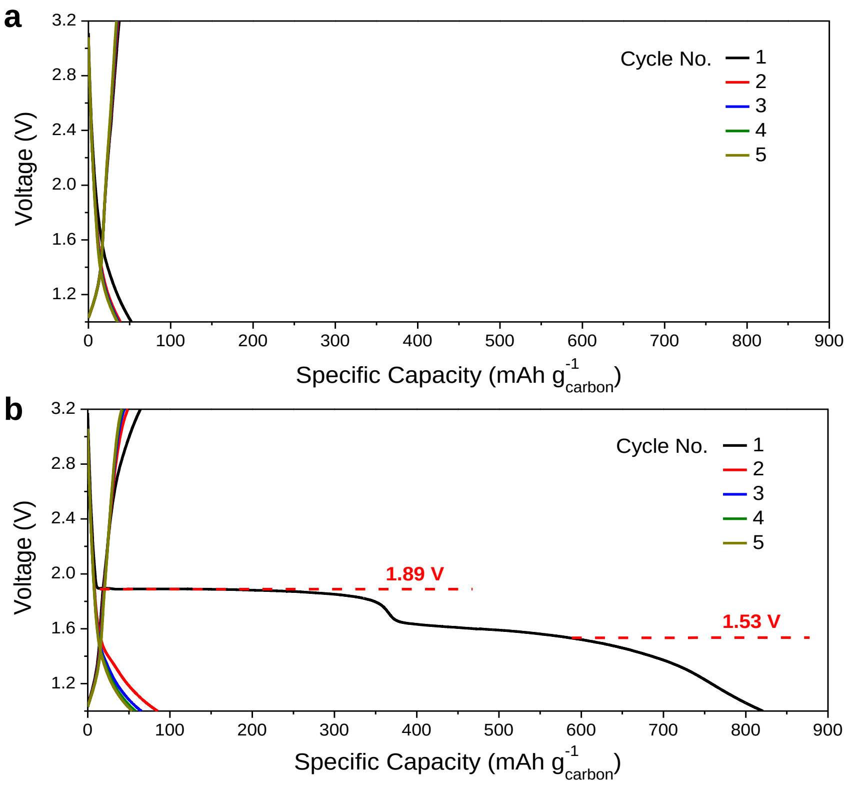

Supplementary Figure S14: Galvanostatic charge-discharge voltage profiles of MWCNTs cycled in (a) $LiNO_3$-free electrolyte and (b) 5 wt% $LiNO_3$-containg electrolyte. In the first cycle, the reduction of $LiNO_3$ results in two additional voltage plateaus at 1.88 and 1.53 V.

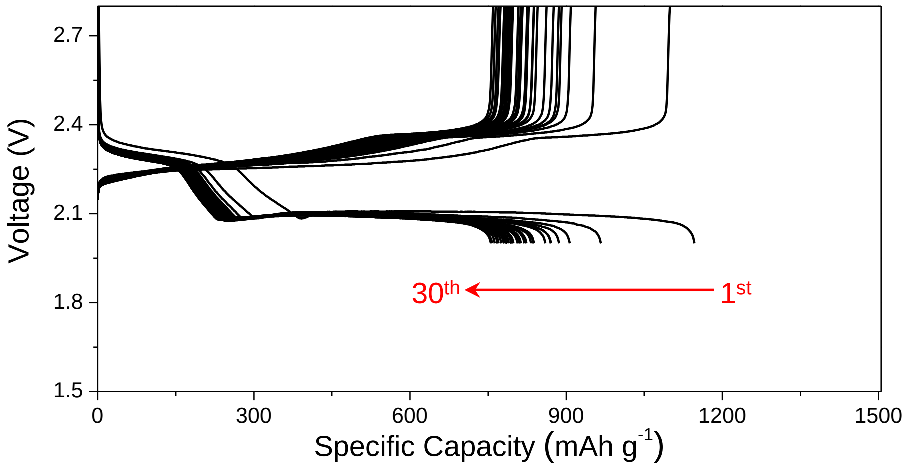

Supplementary Figure S15: Galvanostatic charge-discharge voltage profiles of MWCNTs cycled in the electrolyte with 2 wt% $LiNO_3$. Cut-off voltage: 2.0 ~ 2.8 V.

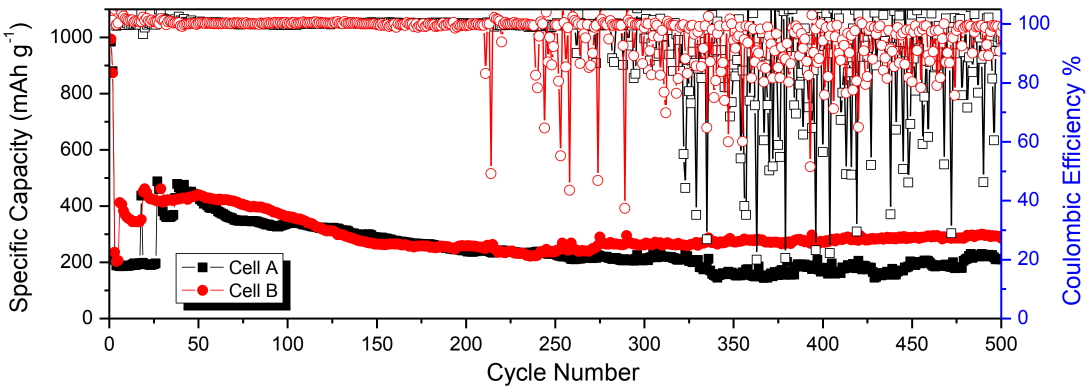

Supplementary Figure S16: Cycling performance of two graphite-sulfur electrodes (Cell A and Cell B) in $LiNO_3$-contained electrolyte at the cycling rate of C/2.5.

References

1. Xu, R.; Li, J. C. M.; Lu, J.; Amine, K.; Belharouak, I. *J. Mater. Chem. A* **2015,** *3*, 4170.

2. Stander, F.; Van Vuuren, C. P. J. *Thermochim. Acta* **1990,** *165*, 85.
